# Crystal structure of human nicotinic acid phosphoribosyltransferase

**DOI:** 10.1016/j.fob.2015.05.002

**Published:** 2015-05-07

**Authors:** Ada Serena Marletta, Alberto Massarotti, Giuseppe Orsomando, Giulio Magni, Menico Rizzi, Silvia Garavaglia

**Affiliations:** a Department of Pharmaceutical Sciences, University of Piemonte Orientale, Largo Donegani 2, 28100 Novara, Italy; b Department of Clinical Sciences, Section of Biochemistry, Polytechnic University of Marche, Via Ranieri 67, 60131 Ancona, Italy

**Keywords:** Na, nicotinic acid, NaAD, nicotinic acid dinucleotide, NAD, nicotinamide adenine dinucleotide, NaMN, nicotinic acid mononucleotide, NamR, nicotinamide riboside, NaPRTase, nicotinic acid phosphoribosyltransferase, NMN, nicotinamide mononucleotide, NMNAT, nicotinamide mononucleotide adenylyltransferase, PRPP, 5-phosphoribosyl-1-pyrophosphate, QA, quinolinic acid, Nicotinic Acid, Preiss–Handler pathway, NAD biosynthesis, Phosphoribosyltransferase, FK866, Recycling NAD pathway

## Abstract

Nicotinic acid phosphoribosyltransferase (EC 2.4.2.11) (NaPRTase) is the rate-limiting enzyme in the three-step Preiss–Handler pathway for the biosynthesis of NAD. The enzyme catalyzes the conversion of nicotinic acid (Na) and 5-phosphoribosyl-1-pyrophosphate (PRPP) to nicotinic acid mononucleotide (NaMN) and pyrophosphate (PPi). Several studies have underlined the importance of NaPRTase for NAD homeostasis in mammals, but no crystallographic data are available for this enzyme from higher eukaryotes. Here, we report the crystal structure of human NaPRTase that was solved by molecular replacement at a resolution of 2.9 Å in its ligand-free form. Our structural data allow the assignment of human NaPRTase to the type II phosphoribosyltransferase subfamily and reveal that the enzyme consists of two domains and functions as a dimer with the active site located at the interface of the monomers. The substrate-binding mode was analyzed by molecular docking simulation and provides hints into the catalytic mechanism. Moreover, structural comparison of human NaPRTase with the other two human type II phosphoribosyltransferases involved in NAD biosynthesis, quinolinate phosphoribosyltransferase and nicotinamide phosphoribosyltransferase, reveals that while the three enzymes share a conserved overall structure, a few distinctive structural traits can be identified. In particular, we show that NaPRTase lacks a tunnel that, in nicotinamide phosphoribosiltransferase, represents the binding site of its potent and selective inhibitor FK866, currently used in clinical trials as an antitumoral agent.

## Highlights

• Human NaPRTase is a functional dimer.
• The structural bases for FK866 lack of inhibition of human NaPRTas were identified.
• Na, Nam and QA phosphoribosyltransferases share a conserved fold.
• Na, Nam and QA phosphoribosyltransferases show distinctive traits in the active site.
• Human and *Enterococcus faecalis* NaPRTase are highly structurally conserved.

## 1 Introduction

**Fig. 1:**
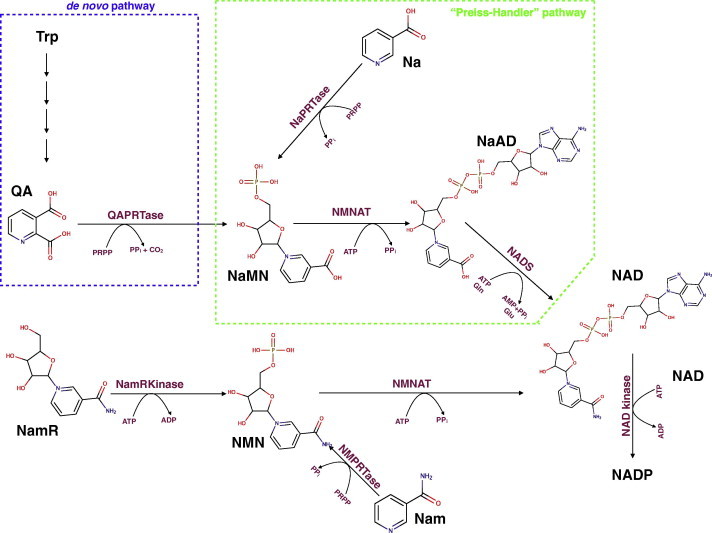
Metabolic pathway for NAD(P) biosynthesis in humans. NAD biosynthesis starting from four different sources of the pyridine ring, namely QA, Na, Nam and NamR, through four distinct ways. The *de novo* pathway (blue dotted line) allows NAD biosynthesis starting from QA derived from Trp and Na is processed to NAD through the Preiss–Handler pathway enzymes (green dotted line). Through a salvage pathway Nam and NamR can be transformed in NMN by NMPRTase and NamRKinase respectively. Finally, some of the cellular NAD can be converted into NADP by NAD kinase (EC 2.7.1.23). QAPRTase, quinolinic acid phosphoribosyltransferase (EC 2.4.2.19); NaPRTase, nicotinic acid phosphoribosyltransferase (EC 2.4.2.11); NMPRTase, nicotinamide phosphoribosyltransferase (EC 2.4.2.12); NamRKinase, nicotinamide riboside kinase (EC 2.7.1.22); NMNAT nicotinamide mononucleotide adenylyltransferase (EC 2.7.7.1); NADS, NAD synthetase (EC 6.3.5.1).

Nicotinamide adenine dinucleotide (NAD) and its phosphorylated form (NADP) are essential ubiquitous coenzymes playing fundamental roles in living cells. Beyond renown redox roles in energy metabolism, NAD(P) is also intimately involved in signaling pathways through a number of consuming reactions that underscore a wealth of physiopathological conditions [1–4]. Indeed, the NAD(P) derivatives nicotinic acid adenine dinucleotide phosphate (NaADP) and cyclic ADP-ribose (cADPR) are among the most potent intracellular calcium-mobilizing agents [4,5]. Moreover, NAD is the substrate for poly(ADP-ribosyl)ation reactions that in higher eukaryotes regulate chromatin function and gene expression, as well as for mono(ADP-ribosyl)ation modifications of target proteins in both mammalian and prokaryotic cells [4]. In all organisms, NAD can also be consumed by important regulatory enzymes, named sirtuins, that catalyze NAD-dependent deacetylation reactions of target proteins [6]. Clearly, physiological NAD depletion caused by overall NAD-consuming reactions necessitates permanent regeneration of this cofactor. Therefore NAD biosynthesis appears of therapeutically value for the treatment of pathological conditions arising from severe altering of NAD(P) homeostasis like in the case of neurological, neoplastic, and infectious disorders, as well as in the process of ageing [1,3,7–10]. Based on current knowledge, four different substrates can be used as a source of the pyridine ring in the NAD biosynthesis: quinolinic acid (QA) in the *de novo* pathway; nicotinic acid (Na), nicotinamide (Nam) and nicotinamide riboside (NamR) in the salvage pathways. While this latter compound is phosphorylated by action of an ATP-dependent kinase activity [11], the three other precursors, QA, Na and Nam, can be individually transferred to a phosphoribosyl pyrophosphate moiety (PRPP) by respective phosphoribosyl-transferase activities [12]. The resulting mononucleotide products, nicotinic acid mononucleotide (NaMN) and nicotinamide mononucleotide (NMN), are then converted to dinucleotide forms, i.e. nicotinic acid dinucleotide (NaAD) and NAD, by action of a single enzymatic activity represented by nicotinamide mononucleotide adenylyltransferase (NMNAT) [13,14]. NaAD is finally amidated to NAD by a NAD synthetase activity (Fig. 1) [7,12,15].

Although the Nam salvaging biosynthetic route appears physiologically the main contributor to keep adequate NAD homeostasis [16–18], the supplementation of Nam does not seem so effective in elevating cellular NAD beyond the basal concentration. Indeed, some mammalian tissues like heart, kidney [19], and red blood cells [20] use preferentially Na for NAD synthesis even in the presence of higher Nam levels physiologically available [21]. Thus, both precursors appear relevant for NAD biosynthesis but with distinct and possibly complementary roles in different tissues [22]. This view is also supported by the observation that human embryonic kidney (HEK293) cell line, when supplemented with Na, but not with Nam, markedly elevated intracellular NAD levels, and showed beneficial effects versus the cytotoxic stress symptoms caused by H_2_O_2_ [22]. This effect of exogenously administered Na likely accounts for at least some effects of this vitamin precursor and suggests novel applications for the treatment of conditions associated with cellular NAD depletion, such as in photodamaged skin [23]. Of note in this context is also the finding that Na is one of the oldest drugs known for its unique anti-lipolytic effects [24], that has been attributed in more recent years to its specific binding to selected receptors present on the plasma membrane of adipocytes [25,26]. In mammals, Na can derive either directly from the diet, or indirectly from the enzymatic deamidation of dietary Nam operated by the gut flora. As shown in Fig. 1, Nicotinate Phosphoribosyltransferase (EC 2.4.2.11) is the first enzyme that catalyzes the synthesis of NaMN and PPi from Na and PRPP. The enzyme, originally named NaMN pyrophosphorylase, was first described by Handler in human erythrocytes, where it plays a key role in elevating NAD levels [20]. In both mice and humans, NaPRTase appears differentially expressed in tissues, being more abundant where Na is the preferential source for NAD biosynthesis [22,27]. Like the bacterial counterpart enzymes, human NaPRTase is strictly specific for Na as a substrate and ADP, NaAD and NAD do not affect its activity [27,28]. The central compounds of cellular metabolism, ATP and Pi, were found to affect human NaPRTase (hNaPRTase) activity. In particular ATP utilization seems to promote allosteric interactions between subunits [27]. Such ATP-driven conformational change can also be inferred by the observation that, in microbial NaPRTs, ATP binding protects from proteolysis [29] and heat inactivation [30].

Although NaPRTase is not a rate-limiting enzyme for NAD synthesis in mammalian cells, several evidences indicate its complementary role in boosting and regulating NAD biosynthesis under certain conditions and in specific local districts [17]. This role appears equally relevant as that proposed for other enzymes of the same pathway, particularly in the context of possible treatments of NAD-related diseases.

Here we report on the crystal structure of the human enzyme in its free form that has been determined at a 2.9 Å resolution. Such structural analysis was instrumental to (i) establish the protein folding conservation among enzymes catalyzing similar phosphoribosyltransferase reactions but using different pyridine substrates, and thus complete the structural information on the NAD salvage pathway enzymes in mammals, (ii) shed further light on the catalytic mechanism of the hNaPRTase and (iii) provide a structural basis for the lack of inhibition on NaPRTase by antitumor drugs targeting NAD biosynthesis metabolism [31]. Moreover, being the first structural report of this enzyme from a mammalian source, it may also assist future structure-based design of effectors of potential medical interest.

## 2 Experimental procedures

### 2.1 Enzyme expression and purification

The procedure adopted for the human gene cloning and bacterial overexpression of recombinant human NaPRTase has been previously reported [28] and here adjusted as follows. Induced cells collected by centrifugation were resuspended in 1/50 original volume with 100 mM KH_2_PO_4_, pH 8, 300 mM KCl, 10 mM imidazole, 1 mM β-mercaptoethanol and 250 units of benzonase nuclease. Bacterial cells membranes were fragmented by applying a high pressure through the use of the French Press twice at 1.5 K Bar. Protease inhibitor cocktail was added (100 μL per 40 mL extraction Buffer) to the crude extract that was clarified by 1 h centrifugation at 18,000 rpm. The supernatant was purified by a His-tag affinity chromatography and a subsequent size-exclusion chromatography. In particular, in the first purification step, the solution containing the soluble fraction of human enzyme was batch-mixed for 30 min with a Qiagen Ni-NTA resin (0.1 mL per mL extract), previously equilibrated with the buffer, and then packed into a chromatographic column. The flow-through and the 30 mM imidazole buffer wash were discarded. The recombinant protein was eluted by two subsequent steps at 250 and 500 mM imidazole. Protein concentration was determined by the Bradford protein assay. Active, SDS–PAGE homogeneous fractions, were pooled, centrifuged and loaded on a Sephacryl S200 16/60 column in a FPLC system. Elution Buffer contained 50 mM Hepes/KOH buffer, pH 7.5, and 300 mM KCl and the flow rate was 0.2 mL/min. Eluted fractions were analyzed by SDS–PAGE. All steps were performed at 4 °C and with 100 μL/40 mL Protease Inhibitor cocktail to preserve the protein stability. The hNaPRTase activity was tested using the assay previously reported [28] and resulted to be 0.27 μmol/min/mg. Using the same assay condition we tested the activity of human NaPRTase in presence of 100 μM FK866 without observing any inhibition of enzyme activity. This procedure allowed us to obtain 20 mg of pure and active human enzyme used for all crystallization trials.

### 2.2 Crystallization and structure determination

Crystals of hNaPRTase were obtained by means of the vapor diffusion technique in sitting drops. 4 μL of a protein solution at a concentration of 15 mg/mL, preincubated with 1 mM Acetyl-CoA and 5 mM DTT, were mixed with an equal volume of a reservoir solution containing 0.1 M Sodium cacodylate pH 6.5 and 1.7 M Sodium acetate and equilibrated against 1.2 mL of the reservoir solution, at 4 °C. Crystals grew to maximum dimension in about 5 days. For X-ray data collection, crystals were quickly equilibrated in a solution containing the crystallization buffer and 20% glycerol as the cryo-protectant and flash frozen at 100 K under a stream of liquid nitrogen. Data up to 2.9 Å resolution were collected at the beamline ID23 EH1 of the European Synchrotron Radiation Facility (ESRF, Grenoble, France). Analysis of the diffraction data set collected allowed us to assign the crystal to the orthorhombic P2_1_2_1_2_1_ space group with cell dimensions *a* = 88.0 Å *b* = 101.1 Å and *c* = 125.1 Å containing two molecules per asymmetric unit with a corresponding solvent content of 49%. Data were processed using the program package XDS [32] and the CCP4 suite of program [33] were used for scaling. The structure determination of hNaPRTase was carried out by means of the molecular replacement technique using the coordinates of a monomer of *Enterococcus faecalis* NaPRTase as the search model (Protein Data Bank ID code 2F7F). Firstly, an improvement of the quality of the search model was carried out by using information contained in the sequence alignment between the hNaPRTase and the *E. faecalis* NaPRTase through the program SCULPTOR [34]. The new procedure Phenix.mr_rosetta [35], that combines crystallographic and structure-modeling algorithms from Phenix and Rosetta, respectively, was used to automatically determine the hNaPRTase structure. Phenix.mr_rosetta returned a unique molecular replacement solution with an LLG of 44.56 and TFZ = 7.4. The initial model was subjected to iterative cycles of crystallographic refinement with the program REFMAC5 [36], alternated with manual graphic sessions for model building using the program Coot [37]. 5% of randomly chosen reflections were excluded from refinement of the structure and used for the Free R factor calculation [38]. The program ARP/warp [39] was used for adding solvent molecules. Refinement was continued until convergence to R factor and Free R factor of 0.19 and 0.24 respectively with ideal geometry. Data collection and refinement statistics are given in Table 1.

### 2.3 Molecular modeling

A molecular dynamics simulation was performed using the GROMACS simulation package v.4.5.3 [40] with the standard GROMOS96 force field. The X-ray crystal structure of hNaPRTase was energy-minimized *in vacuo*, using 1000 steps of steepest descent in order to remove distorted geometries and with the protein embedded in a solvent box. The next step was a 2 ns simulation with position restraints to the protein, followed by a 500 ps unrestrained simulation. The last 500 ps of the simulation were used to compute an average structure for hNaPRTase, which, after minimization with 1000 steps of steepest descent, represents the final model. After aminoacids stereochemistry has been assessed with the program PROCHECK [41], the model was used for docking simulations. The ligands tested for docking were FK866, Na, NaMN, Na plus PRPP and ATP. Ligand structures were built from a SMILES string and were minimized using Omega2 [42]. The docking simulations were performed using FRED, and using the default settings [43]. Each ligand-NaPRTase complex was subsequent subjected to a molecular structure optimization using SZYBKI [44] in order to refine the molecular structure using the Merck Molecular Force Field with solvent effect.

### 2.4 Deposition

The atomic coordinates and structure factors of human NaPRTase have been deposited with the Protein Data Bank (www.rcsb.org) with accession code: 4YUB.

### 2.5 Illustrations

Figures were generated by using the program pymol [45].

## 3 Results and discussion

### 3.1 Overall quality of the model

**Fig. 2:**
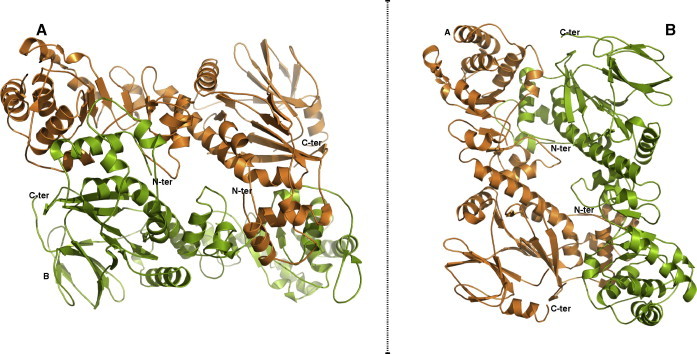
Overall oligomeric structure of human NaPRTase. The enzyme has a dimeric structure and monomers A and B are colored in orange and green, respectively. Two different orientations of the dimer, showing the head-to-tail arrangement of the monomers, are represented. N-ter, N-terminus; C-ter, C-terminus.

The three-dimensional structure of hNaPRTase has been solved by molecular replacement and refined at a resolution of 2.9 Å. The crystal asymmetric unit contains a dimer of hNaPRTase and a total of 98 solvent molecules. The two protein molecules have been designated as monomers A and B (Fig. 2). Monomer A contains 501 residues out of 538 and no electron density is present for the following regions: first 15 residues at the N-terminus, residues 379–389, residues 409–415, and last five residues at the C-terminus. Monomer B contains 501 residues out of 538 and no electron density is present for the following regions: first 15 residues at the N-terminus, residues 379–389, residues 408–414, and last four residues at the C-terminus. The stereochemistry of the model has been assessed with the program PROCHECK [41]: 99% of protein residues fall in the favoured regions of the Ramachandran plot with no outliers.

### 3.2 Overall structure of human NaPRTase monomer

**Fig. 3:**
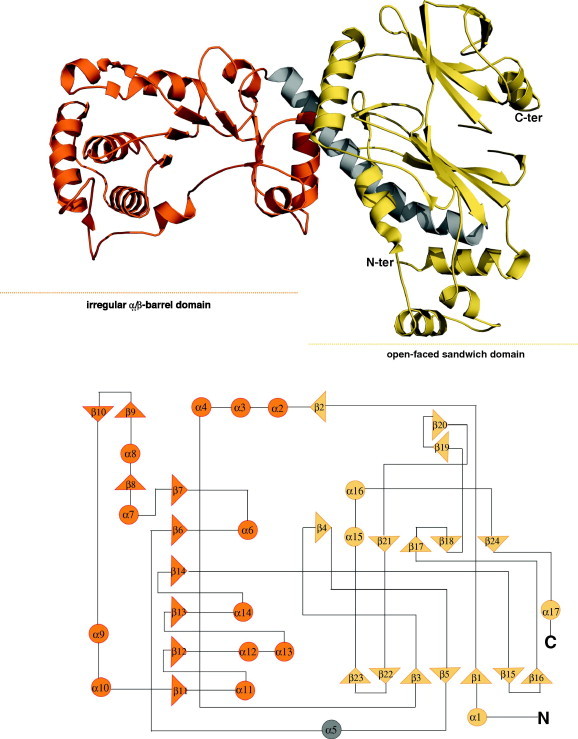
Overall monomer structure of human NaPRTase. Ribbon representations of the overall structure of hNaPRTase monomer. Each monomer consists of two domains: the “open-faced sandwich domain” is shown in yellow, while the “irregular α/β barrel domain” is colored orange. The two domains are connected by a long α-helix, colored in gray. The topology model of the monomer highlight that the “open-face sandwich domain” is composed of residues by both the N- and C-terminal regions and the “irregular barrel domain” contains a six-stranded α/β core.

Human NaPRTase monomer folds into 17 α-helices, 24 β-strands and the connecting loops organized in two domains: a first domain characterized by an irregular α/β barrel and a second open-faced sandwich domain (Fig. 3). In particular, as shown in the protein topology diagram in Fig. 3, the open-face sandwich domain is composed of residues provided by both the N- and C-terminal regions of the protein (residues 16–127 and 390–534). It consists of 4 α-helices disposed like a shield and delimiting the open space where 15 β-strands form 2 antiparallel β-sheets, showing a specular two-by-two arrangement. Indeed, the seven-stranded fully antiparallel β-sheet (β1, β3, β5, β15, β16, β22 and β23) and the four-stranded partially antiparallel β-sheet (β17, β18, β21 and β24) face each other. The other two smaller β-sheets (β2, β4 and β19, β20) delimit the domain. The longest helix α5, with 8 turns (residues 128–159) connects the two domains. The irregular α/β barrel domain (residues 160–378) contains a six-stranded α/β core (β6, β7, β11, β12, β13, and α6, α11, α12, α13, α14) that identifies hNaPRTase as a member of the type II Phosphoribosyltransferases (PRTases) subfamily. The two monomers present in the asymmetric unit have the same conformation showing a r.m.s. deviation of 0.5 Å after superposition based on all Cα atoms. The hNaPRTase structural organization turns out to be highly similar to that of *E. faecalis* NaPRTase, used as a model for the molecular replacement. Indeed, the two structures can be optimally superimposed with a r.m.s.d. of 2.4 Å based on 482 equivalent Cα atoms. As could be expected, the overall fold is highly conserved with two domains connected by a long α-helix. Moreover, in spite of the low sequence identity (34%) the active site residues are conserved in the two enzymes revealing an identical mode of binding for substrates. In particular, Y17, H205, R262, S206 K333 R163 and T162 that in *E. faecalis* NaPRTase defines the enzyme active site are strictly conserved in hNaPRTase with equivalent positions occupied by Y21, H213, R318, S214 K396 R171 and L170. The high degree of structural conservation observed between the human and the bacterium NaPRTases could be due to an evolutionary adaptation. Indeed, *E. faecalis* is a commensal bacterium inhabiting the gastrointestinal tracts of humans where, in the small intestine, Na is a preferred source for NAD synthesis.

### 3.3 The dimer of human NaPRTase

hNaPRTase has a predicted molecular weight of 58 KDa and it has been reported to show a native molecular mass of about 87,000 Da by gel filtration [46,28]. The crystal structure of hNaPRTase reveals the presence of an intimately associated dimer in the asymmetric unit with 3000 Å^2^ of the surface area buried at the dimer interface. The two monomers are arranged head to tail with the N-terminal domain in one monomer contacting the α/β barrel in the other monomer (Fig. 2). Therefore our structural data confirm that the minimal functional unit in the hNaPRTase enzyme consists of a dimer as already proposed for *Thermoplasma acidophilum* NaPRTase [47]. Moreover, the major interactions established at the dimeric interface in *Ta*NaPRTase were observed to be of ionic rather than hydrophobic nature [47]. Our structural analysis confirmed that the main interactions between monomers are of ionic nature also in hNaPRTase.

### 3.4 The active site

**Fig. 4:**
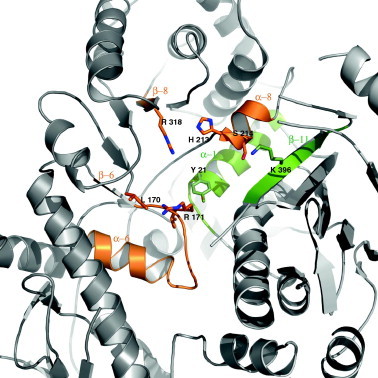
Active site representation of human NaPRTase. Ribbon representation of the active site formed at the dimeric interface, between the α/β barrel domain of monomer A, colored in orange, and the monomer B, shown in green. Side chains of key residues involved in catalysis are shown.

**Fig. 5:**
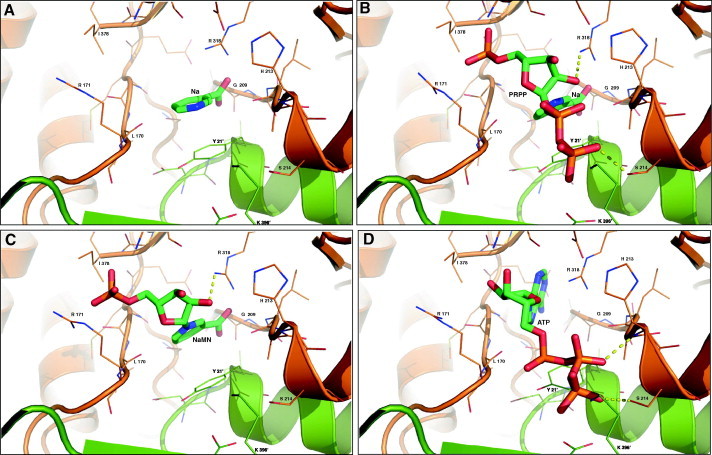
Molecular docking of the hNaPRTase active site dimeric interface in complex with different ligands. Side chains of residues participating in the binding ligands ((A) Na; (B) NaMN; (C) Na and PRPP; (D) ATP) are represented as thin sticks and their identity is indicated, whereas ligands are depicted as green sticks. Hydrogen bonds are shown as yellow dotted lines.

Since we have been unable to obtain crystals of hNaPRTase in complex with any ligand, the binding mode of substrate/product to the enzyme active site was investigated through molecular docking simulations. The functional dimeric model of hNaPRTase was first re-built using the X-ray crystal structure and subsequently refined by molecular dynamics simulations. Na, NaMN, Na plus PRPP, and ATP were docked in the generated model. As already reported in the previously characterized NaPRTases [47], the active site consists of a pocket located at the dimer interface. In particular, in hNaPRTase it is delimited by the loops connecting α-helix 6 with β-strand 6 and α-helix 8 with β-strand 8 of α/β barrel domain of monomer A and completed by residues of β-strand 11 and α-helix 1 from monomer B (Fig. 4). According to our modeling procedure the pyridine ring of Na establishes contacts with the protein environment that involve a π–π stacking interaction with Y21B and Van der Walls contacts with L170A, G209A and L211A. In addition, the carboxyl group of Na is engaged in a strong ionic interaction with R318A (Fig. 5a). It has previously been reported that the G201 to Ala substitution causes a 90% reduction of enzyme activity even in the presence of ATP and that the Y21 to Ala substitution results in the complete loss of activity in absence of ATP and in a 50% reduction in the presence of ATP [28]. Our data are therefore in agreement with the functional analysis carried out by site directed mutagenesis and provide a solid structural explanation for the role played by these residues in catalysis. We then investigated the mode of binding for Na and PRPP when simultaneously bound to the enzyme active site (Fig. 5b). PRPP appears to be stabilized by two ionic interactions with R171A and K396B that are engaged in a salt bridge with the phosphate and pyrophosphate moieties, respectively. PRPP is further stabilized by two hydrogen bonds established between its ribose oxygen and R318A and between the pyrophosphate moiety and S214A. Our observations confirm the functional analysis carried out by site-directed mutagenesis previously reported in which the replacement of R318 with Ala completely abolish the enzyme activity in absence of ATP while a reduction of 50% is observed in the presence of ATP [28]. The ATP binding mode was also investigated and turned out to be quite similar to that observed for PRPP (Fig. 5d). In particular the ATP γ phosphate group is salt bridged to K396B, one of the key residue for PRPP binding, while the β phosphate group is involved in hydrogen bonds with H213A (backbone amide) and S214A. The adenine moiety of ATP is involved in a π-polar interaction with R318A. Also in this case, our observations remark an important role in catalysis for H213 that when mutated to Ala was reported to cause a 50% reduction of the enzyme activity [28]. Finally, the binding mode of the product NaMN was investigated (Fig. 5c). As expected, the orientation of both the Na and phosphate moieties in the mononucleotide, is identical to what observed for the corresponding chemical entities in the model of the Na-PRPP enzyme complex. In conclusion, our structural models for substrate binding provide a solid explanation for the role played in catalysis by residues that were previously reported to be essential for enzyme activity by a functional analysis based on site directed mutagenesis [28]. The perfect coherence between biochemical data reported in previous study and our structural observations, also proves that our molecular docking simulation are robust. Therefore, besides R318, Y21, H213 that were already subjected to site directed mutagenesis and that we confirm to be essential residues for catalysis, we propose that R171, K396 and S214 also play an essential role in substrate recognition. Moreover, we identify R318 as the major player in catalysis. Indeed, this residue is involved in the binding of all substrates by recognizing different chemical entities: the carboxylic moiety of Na, the ribose ring of PRPP and the adenine base of ATP. In addition, our *in silico* model of the complex hNaPRT-Na and hNaPRT-NaMN allows a detailed structural comparison of the mode of binding of Na, Nam and QA in the three phosphoribosyltransferases. At the level of the active sites we observe a high degree of structural conservation mainly between hNaPRTase and hNMPRTase, while a less significant conservation is observed with hQAPRTase. However, the mode of binding of all the three different pyridines is conserved, while different is the protein milieu surrounding the substrate. In particular, hQAPRTase provides a more positively charged environment that is required for the recognition of QA that carries two negatively charged carboxylic groups. Although our structural analysis does not disclose any obvious common features for the catalytic mechanism adopted by hQAPRTase, hNMPRTase and hNaPRTase, a conserved mode of binding of the pyridine ring containing substrate clearly emerges. More information can be extracted if the structural analysis is confined to hNaPRTase and hNMPRTase. In these two enzymes, the mode of binding of Na and Nam appears highly similar. Indeed, the pyridine ring is sandwiched by an aromatic/hydrophobic clamp in both enzymes: Y18/F193 in hNMPRTase and Y21/L170 in hNaPRTase. R311, R196 and K400 that in hNMPRTase play a key role in the binding of PRPP, are strictly conserved in hNaPRTase (R318, R171 and K396). On the other hand, a specific residue appears to participate in guaranteeing the strikingly substrate specificity displayed by hNMPRTase and hNAPRTase for nicotinamide versus nicotinic acid. While in hNMPRTase D219 interacts with the amide moiety of Nam, in hNaPRTase the structurally equivalent position is occupied by S214 contributing to direct the enzyme specificity toward nicotinate. Other structural determinants that do not clearly emerge in our docking models are however required to fully explain the strict specificity toward nicotinic acid displayed by hNaPRTase. Overall, a conserved mode of binding of QA, Na and Nam in the respective phosphoribosyltransferase is observed. However, the structural architecture of the active site is sensibly different in hQAPRTase and peculiar traits also feature hNMPRTase and hNaPRTase. Therefore, we propose that the wealth of structural information that is now available for all the three enzymes can be successfully exploited for the design of highly selective inhibitors.

### 3.5 Comparison with hNMPRTase and hQAPRTase

**Fig. 6:**
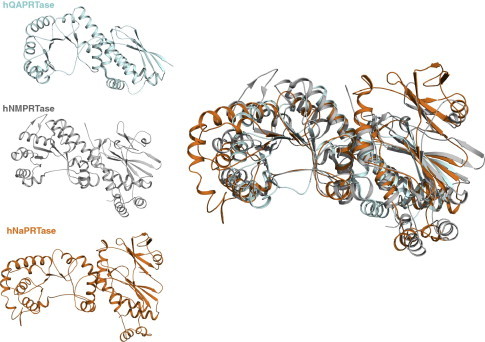
Structural comparison of the three human Type II phosphoribosyltransferases involved in NAD biosynthesis. Ribbon representations of human QAPRTase in blue, NMPRTase in gray and NaPRTase in orange are shown separately (*left panel*). The structural superposition of the three enzymes is also represented (*right panel*).

Despite sharing very limited sequence similarity, hNaPRTase shows a molecular fold that closely resembles those firstly described for hNMPRTase [31,48] and hQAPRTase [49] (Fig. 6). This fact is in accordance with their common function, consisting in the transfer of the phosphoribosyl group from PRPP onto their respective substrates. A DALI search shows a significant similarity with hNMPRTase with a *Z*-value of 22.2 and a r.m.s. deviations of 3.6 Å based on superposition of 463 equivalent Cα atoms. hQAPRTase is the smallest in size among the three PPRTases, in agreement with the hypothesis that it is the most ancient [50]. At the contrary, hNaPRTase contains 46 and 241 more amino acid residues than hNMPRTase and hQAPRTase, respectively. The structural superposition of the three enzymes highlights how some of the additional residues in hNaPRTase form two structural regions that, from a Blastp search, appear to be univocally present in mammal NaPRTases (Fig. 6). The first unique region spans amino acids 223–264 and contains two β-strands (β9–β10) and one α-helix (α9) that extend the borders of the α/β barrel domain and contribute to contact the other monomer (Fig. 3). Indeed, the side chain of D227 is hydrogen bonded to the side chain of R31 of the opposite monomer. The second unique region (447–472) of hNaPRTase encompasses three β-strands (β19, β20 and β21) that stand at the bottom of the open-faced sandwich (Fig. 3). These two peculiar regions in hNaPRT are located on the enzyme surface and do not have any structural impact on the enzyme active site. Therefore, there is no evidence of their involvement in catalysis or substrate binding. On the other hand, since the activity of extracellular hNMPRTase as a potent cytokine is well documented [51], we speculate that the structural variation of the protein surface may sustain still unknown functions of an extracellular form of hNaPRTase. The analysis of conserved versus peculiar traits in the active site of the three human phosphoribosyltransferases has been carried out based on optimal structural superposition. The active site of hNAPRTase and hNMPRTase shows a high degree of structural conservation that appears necessary for the binding of the common substrate PRPP and the highly similar substrates Na and Nam. Indeed, for the latter case, the pyridine ring recognition is guaranteed by few strictly conserved residues; in particular, Y21, H213, R318 in hNaPRTase appear to be structurally equivalent to Y18, H247 and R311 in hNMPRTase; on the other hand, F193 that in hNMPRTase provides an aromatic stacking interaction with the Nam pyridine ring is substituted by L170 in hNaPRTase. Of note is the observed different conformation of another strictly conserved residue located in the enzyme active site, i.e. R196 in hNMPRTase that is equivalent to R171 in hNaPRTase. In the latter, the positively charged side chain closes up the enzyme active site and is one of the structural determinants hampering binding of FK866 to hNaPRTase. When we included in the structural analysis hQAPRTase, a lower degree of structural conservation amongst all the three enzymes, emerged. First, the active site in hQAPRTase is sensibly more positively charged with residues R102, R138, R161, K139 and K171 all involved in the binding of QA that carries two carboxylic groups. Among all these residues R102 appears to be strictly structurally conserved in all the three phosphoribosyltransferase occupying the equivalent position of R196 in hNAMPTase and R171 in hNaPRTase. This residue interacts with the functional moiety present in position 3 of the pyridine ring in all the three different ligands: QA, Na and Nam. On the other hand, R138 and R161 in hQAPRTase appear as a peculiar feature of this enzyme and interact with the second carboxyl group present only in the structure of QA, in position 2 of its pyridine ring.

### 3.6 Lack of inhibition by FK866

**Fig. 7:**
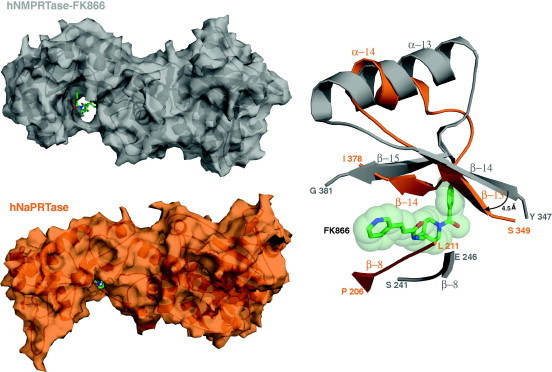
FK866-binding-site: structural comparison between hNMPRTase and hNaPRTase. Human NMPRTase structure in complex with its inhibitor FK866 (PDB ID: 2GVJ) is colored in gray and hNaPRTase is colored in orange. Structural superposition between these two structures highlight, in hNaPRTase, of the tunnel where FK866 binds in hNMPRTase. Buried surface areas of hNMPRTase (*upper left panel*) and hNaPRTase (*lower left panel*) monomers are represented and FK866 is shown as green stick. A ribbon represented close-up view (*right panel*) of the superposed secondary structure elements sterically hampering (in hNaPRTase) or permitting (in hNMPRTase) the binding of FK866. Steric hindrance of FK866 is represented by light green spheres.

FK866 is a potent hNMPRTase competitive inhibitor endowed with a strong antitumor activity and is currently undergoing clinical trials for the treatment of cancer [31]. Surprisingly, FK866 does not inhibit hNaPRTase [31] and an explanation of this unexpected observation is still missing. The inhibitory mechanism of action on the target has been investigated by structural studies [31]. FK866 binds to hNMPRTase in a tunnel observed at the dimer interface and defined by the parallel β sheet of the α/β barrel domain of each monomer [31]. In particular, two small β strands (β14 and β15) that participate in shaping the tunnel and are located in close proximity of the enzyme’s active site, contribute to the recognition and binding of FK866. In hNaPRTase the corresponding region (β13 and β14) revealed a different orientation determined by a 10 residues deletion that shorten the preceding alpha helix (α14). The resulting structural arrangement sterically prevents the binding of FK866 to hNaPRTase due to severe clashes between the inhibitor and the protein region defining the tunnel (Fig. 7).

## 4 Conclusion

NaPRTase is the first enzyme in the Preiss–Handler NAD salvage pathway and therefore has an important role in NAD metabolism, which is involved in a large number of physiological and pathological conditions in all organisms. Beyond its role in NAD biosynthesis, it was hypothesized that, in humans, NaPRTase could be linked to other central metabolic pathways [27] as suggested also for hACMSD, an enzyme regulating the *novo* NAD synthesis [52]. Several studies have underlined the NaPRTase importance for NAD homeostasis in mammals, but no crystallographic data are available for higher eukaryotes. Our investigations reveal a high degree of structural conservation at the level of the overall fold with both bacterial NaPRTase and hNMPRTase. However, while in the case of the bacterial enzyme the structural similarity extend also to the active site, significant differences emerge in such a region with hNMPRTase. Indeed, we confirm that the functional unit of hNaPRTase is a dimer with the active site located at the domains interface in each monomer, as observed in hNMPRTase. On the other hand, the structural organization of the active site in the two enzymes, clearly shows peculiar traits that explain both the strikingly different substrate specificity as well as the specific inhibitory action of FK866 on hNMPRTase. hNaPRT is a medically relevant enzyme; its expression levels have been shown to vary consistently among populations and in different pathological conditions [53–56] including cancer where variants of the enzyme have also been identified [54]. Moreover, it has recently been reported that the adverse effects caused by reactive oxygen species induced by the treatment with the anticancer hNMPRTase inhibitors (FK866 and analogues), can be reduced by a co-treatment with nicotinic acid that is linked to the over-expression of hNaPRTases [57]. Indeed, the administration of nicotinic acid protected non-tumor cells from toxic effects resulting from depletion of NAD caused by hNMPRTase inhibition [57]. Therefore, a potential future effective antitumor therapy targeting NAD biosynthesis should be featured by both a strong blockage of hNMPRTase to obtain high toxicity toward tumor cells and no inhibition of hNaPRTase in order to reduce the toxic effect of the anticancer drug on non-tumoral cells. The availability of the crystal structure of both hNMPRTase and hNaPRTase is of high relevance for the structure-based rational design of potent and selective, i.e. acting on hNMPRTase only, inhibitors of potential medical relevance. In conclusion our structural data may find application for cancer treatment and may help in elucidating the complex and yet unclear interconnections among distinct metabolic pathways in humans.

## Contributions

S.G., A.S.M., G.O., A.M. and G.M. performed experiments and collected and analyzed the data; S.G. designed the research; S.G. and M.R. wrote the paper.

## Competing financial interests

The authors declare no competing financial interests.

## Figures and Tables

**Table 1:** Data collection and refinement statistics.

	hNaPRTase crystal
*Data collection*	
Space group	P2_1_2_1_2_1_
Cell dimensions	
*a*, *b*, *c* (Å)	88.0, 101.1, 125.1
*α*, *β*, *γ* (°)	90, 90, 90
Resolution (Å)	2.9
*R*_pim_/*R*_merge_	6.1 (20.5)/11.4 (37)
Mean(I)/sd(I)	10.0 (3.7)
Completeness (%)	99.7 (99.3)
Redundancy	4.4 (4.2)

*Refinement*	
Resolution (Å)	2.9
No. reflections	25,261 (2464)
*R*_work_/*R*_free_	0.19/0.24
No. atoms	
Protein	7576
Water	98
Mean B-factors	
Protein (Å^2^)	42.67
Water (Å^2^)	33.60
R.m.s deviations	
Bond lengths (Å)	0.014
Bond angles (°)	1.83
